# Gorlin syndrome-derived induced pluripotent stem cells are hypersensitive to hedgehog-mediated osteogenic induction

**DOI:** 10.1371/journal.pone.0186879

**Published:** 2017-10-31

**Authors:** Daigo Hasegawa, Hiromi Ochiai-Shino, Shoko Onodera, Takashi Nakamura, Akiko Saito, Takeshi Onda, Katsuhito Watanabe, Ken Nishimura, Manami Ohtaka, Mahito Nakanishi, Kenjiro Kosaki, Akira Yamaguchi, Takahiko Shibahara, Toshifumi Azuma

**Affiliations:** 1 Department of Oral and Maxillofacial Surgery, Tokyo Dental College, Tokyo, Japan; 2 Department of Biochemistry, Tokyo Dental College, Tokyo, Japan; 3 Laboratory of Gene Regulation, Faculty of Medicine, University of Tsukuba, Ibaraki, Japan; 4 Biotechnology Research Institute for Drug Discovery, National Institute of Advanced Industrial Science and Technology (AIST), Tsukuba, Ibaraki, Japan; 5 Center for Medical Genetics, Keio University School of Medicine, Tokyo, Japan; 6 Oral Health Science Center, Tokyo Dental College, Tokyo, Japan; National Cancer Center, JAPAN

## Abstract

Gorlin syndrome is an autosomal dominant inherited syndrome that predisposes a patient to the formation of basal cell carcinomas, odontogenic keratocysts, and skeletal anomalies. Causative mutations in several genes associated with the sonic hedgehog (SHH) signaling pathway, including *PTCH1*, have been identified in Gorlin syndrome patients. However, no definitive genotype—phenotype correlations are evident in these patients, and their clinical presentation varies greatly, often leading to delayed diagnosis and treatment. We generated iPSCs from four unrelated Gorlin syndrome patients with loss-of-function mutations in *PTCH1* using the Sendai virus vector (SeVdp(KOSM)302). The patient-derived iPSCs exhibited basic iPSC features, including stem cell marker expression, totipotency, and the ability to form teratomas. *GLI1* expression levels were greater in fibroblasts and patient-derived iPSCs than in the corresponding control cells. Patient-derived iPSCs expressed lower basal levels than control iPSCs of the genes encoding the Hh ligands Indian Hedgehog (IHH) and SHH, the Hh acetyltransferase HHAT, Wnt proteins, BMP4, and BMP6. Most of these genes were upregulated in patient-derived iPSCs grown in osteoblast differentiation medium (OBM) and downregulated in control iPSCs cultured in OBM. The expression of *GLI1* and *GLI2* substantially decreased in both control and patient-derived iPSCs cultured in OBM, whereas *GLI3*, *SHH*, and *IHH* were upregulated in patient-derived iPSCs and downregulated in control iPSCs grown in OBM. Activation of Smoothened by SAG in cells grown in OBM significantly enhanced alkaline phosphatase activity in patient-derived iPSCs compared with control iPSC lines. In summary, patient-derived iPSCs expressed lower basal levels than the control iPSCs of the genes encoding Hh, Wnt, and bone morphogenetic proteins, but their expression of these genes strongly increased under osteogenic conditions. These findings indicate that patient-derived iPSCs are hypersensitive to osteogenic induction. We propose that Hh signaling is constituently active in iPSCs from Gorlin syndrome patients, enhancing their response to osteogenic induction and contributing to disease-associated abnormalities.

## Introduction

Gorlin syndrome, also referred to as basal cell nevus syndrome or nevoid basal cell carcinoma syndrome, is an autosomal dominant inherited syndrome that predisposes a patient to the formation of basal cell carcinomas (BCCs) and odontogenic keratocysts as well as to skeletal anomalies such as increased bone mass. The skeletal changes that characterize Gorlin syndrome were first described by Gorlin and Goltz in 1960 as an autosomal dominant syndrome in a family predisposed to BCCs, jaw cysts, and bifid ribs [1].

Patched-1 (PTCH1) is a Hedgehog (Hh) receptor that acts as a negative regulator of constitutive Hh signaling by preventing the G protein-coupled receptor Smoothened (SMO) from entering the cilium in the absence of Hh protein binding. The binding of PTCH1 with Hh proteins facilitates SMO access to the cilium, thereby promoting the release of GLI family transcription factors from a multi-protein complex. GLI proteins then enter the nucleus, where they regulate the transcription of target genes. Causative mutations in several genes associated with the sonic hedgehog (SHH) signaling pathway, including *PTCH1* [2–7], *PTCH2* [7], and *SUFU* [8], have been identified in Gorlin syndrome patients. A previous study reported that *Ptch1*-deficient (*Ptch1*^+/−^) mice and Gorlin syndrome patients exhibited increased bone mass as adults, suggesting that the patients’ stem cells were hypersensitive to osteogenic induction [9]. However, no definitive genotype—phenotype correlations are evident in Gorlin syndrome patients, and there is a high degree of variability in symptom presentation [10–12]. The heterogeneity and relative rarity of this disease have confounded the elucidation of its pathogenesis. In addition, its heterogeneous nature can lead to delayed diagnosis and treatment, thereby increasing morbidity and mortality.

Disease-specific stem cells offer an unprecedented opportunity to recapitulate both normal and pathological human tissue formation *in vitro*, enabling the investigation of disease pathogenesis and facilitating the development of new treatments. A tractable method for establishing immortal pluripotent stem cell cultures from diseased individuals would also provide a foundation for developing autologous cell therapies that evade immune rejection and allow for the correction of genetic defects prior to tissue reconstitution [12–16].

The aim of this study was to elucidate the pathophysiology of Gorlin syndrome-associated tumorigenesis and skeletal abnormalities. To do this, we generated induced pluripotent stem cells (iPSCs) from oral fibroblasts (OFs) isolated from four Gorlin syndrome patients using the Sendai virus vector SeVdp(KOSM)302L. All four patients exhibited *PTCH1* mutations, as determined by next-generation exome sequencing [17]. We found that patient-derived OF iPSCs (G-OFiPSCs) expressed lower basal levels of Hh genes, Wnt genes, *BMP4*, and *BMP6* compared with control cells. However, osteogenic activation in response to the SMO activator SAG was enhanced in G-OFiPSCs compared with control cells, suggesting that G-OFiPSCs are hypersensitive to osteogenic induction. This is the first study to recapitulate a cellular phenotype consistent with the clinical features of Gorlin syndrome (i.e., osteogenic hypersensitivity) *in vitro*. Disease-specific iPSCs may provide a valuable model for analyzing the pathogenesis of Gorlin syndrome and investigating novel treatments.

## Materials and methods

### Cell culture

Gorlin syndrome oral fibroblasts (G-OFs) were obtained from oral tissues resected from four Gorlin syndrome patients undergoing treatment. All four patients provided written informed consent for the use of their tissue samples, and the genomic analyses were approved by the Ethics Committee of Tokyo Dental College (no. 527 and no. 575). The KD human lip fibroblast (JCRB9103; JCRB Cell Bank, Osaka, Japan) was used as a control. All human fibroblasts were cultured in Dulbecco’s Modified Eagle’s Medium (DMEM) (Invitrogen, Carlsbad, CA, USA) supplemented with 10% fetal bovine serum (FBS) (Invitrogen) and 1% penicillin/streptomycin (Invitrogen). Fibroblasts were maintained at 37°C in an atmosphere with 5% CO_2_ and seeded on 100 mm dishes. The culture medium was replaced every 2 days. The control human iPSC lines 201B7 (HPS0002) [18] and Nips-B2 (HPS0223) [19] were purchased from Riken Bioresource Center (Tsukuba, Japan).

### Generation of human iPSCs

To generate Gorlin syndrome iPSCs, we transfected 1 × 10^5^ G-OF cells in 12-well plates with the Sendai virus vector SeVdp(KOSM)302L [20]. On day 28, the resulting iPSC colonies were selected and plated onto SNL76/7 feeder cells in 24-well plates. These feeder cells had been clonally derived from mouse fibroblast STO cells transformed with neomycin-resistance genes and murine leukemia inhibition factor genes. The iPSCs were maintained with SNL76/7 feeder cells in human ES medium (DMEM supplemented with F-12 nutrient mixture [DMEM/F-12]; Invitrogen) with 20% knockout serum replacement (Invitrogen) supplemented with 1 × nonessential amino acids solution (Chemicon, Temecula, CA), 2 mM L-glutamine (Chemicon), 0.11 mM 2-mercaptoethanol (Wako Pure Chemical Industries Ltd., Osaka, Japan), 1% penicillin/streptomycin (Invitrogen), and 5 ng/ml human basic fibroblast growth factor (bFGF; ReproCELL Inc., Yokohama, Japan). The culture medium was replaced every day until the cells reached confluence.

### Embryoid body formation

SNL76/7 feeder cells were carefully removed using CTK dissociation solution (ReproCELL Inc., Yokohama, Japan). The plates were rinsed twice with PBS, and the iPSCs were dissociated using a cell scraper and transferred to low-attachment Petri dishes to generate embryoid bodies (EBs) EBs were maintained in human ES medium without bFGF for 21 days. The culture medium was replaced every 3 days.

### Teratoma formation

The human iPSC colonies were removed from the culture plates using a cell scraper. The cells (1 × 10^6^ cells in 20 μl PBS) were injected into the testes of 8–10-week-old male C.B-17 SCID mice (Charles River Laboratories Japan, Inc., Yokohama, Japan). Teratomas were collected 9–12 weeks after the injections and fixed with 10% neutral buffered formalin for 24 h, paraffin-embedded, and sectioned for histological assays. All of the mouse studies were conducted in accordance with protocols approved by the Animal Research Committee of Tokyo Dental College (No. 270401).

### SMO inhibitor and SMO agonist treatment

Control fibroblasts (KD cells) and G-OFs were seeded at a density of 0.4 × 10^5^/well on 12-well plates in DMEM supplemented with 10% FBS for 24 h. The culture medium was replaced with DMEM supplemented with 0.5% FBS (serum starvation conditions) for 48 h. The fibroblasts were incubated for 48 h in the presence or absence of the SMO inhibitor cyclopamine (100 nM; LKT Laboratories, Inc., MN, USA), the SMO agonist SAG (1 μM; Merck Millipore, Darmstadt, Germany), or both.

The EBs were maintained in suspension culture with bFGF-free human ES medium for 6 days and then cultured in bFGF-free human ES medium supplemented with 2 μM thiazovivin (Wako Pure Chemical Industries Ltd.) for 1 h at 37°C. Then, the EBs were collected and dissociated from the culture plates using 0.5 mg/ml collagenase type IV (Wako Pure Chemical Industries Ltd.) for 20 min at 37°C and subsequently incubated with 0.05% trypsin—EDTA (Invitrogen) for 5 min at 37°C. The trypsinized EBs were seeded on cell culture dishes at a density of 1.8 × 10^4^ cells/cm^2^ in DMEM supplemented with 10% FBS for 24 h. The culture medium was replaced with DMEM supplemented with 0.5% FBS (serum starvation conditions) prior to treatment for 48 h with or without 100 nM cyclopamine, 1 μM SAG, or both.

### Osteogenic differentiation

We previously reported an effective method for inducing osteogenesis in human iPSCs [21,22]. Briefly, human iPSCs were incubated in the presence or absence of a SMO agonist or inhibitor in osteoblast differentiation medium (OBM). The OBM consisted of α-MEM (Invitrogen) supplemented with 10% FBS, 50 μg/ml L-ascorbic acid (Wako Pure Chemical Industries Ltd.), 10 mM β-glycerophosphate (Wako Pure Chemical Industries Ltd.), and 10 nM dexamethasone (Wako Pure Chemical Industries Ltd.). A combination of cytokines, referred to as FIT and comprising 25 ng/ml bFGF, 1 ng/ml TGF-β1 (Wako Pure Chemical Industries Ltd.), and 100 ng/ml IGF-1 (Wako Pure Chemical Industries Ltd.), was added on the following day (day 0). The iPSCs were cultured for an additional 10 days, with the OBM replenished every 3 days.

### Protein extraction and immunoblotting

The cells were lysed with RIPA buffer (25 mM Tris-HCl at pH 7.5, 150 mM NaCl, 1% Halt protease inhibitor cocktail, 1% sodium deoxycholate, 1% sodium lauryl sulfate, and 1% NP-40). Equivalent protein concentrations were separated by sodium dodecyl sulfate polyacrylamide gel electrophoresis (SDS-PAGE) and transferred to a polyvinylidene difluoride (PVDF) membrane (Immobilon-P, Millipore, Bedford, MA). Nonspecific binding was blocked with Blocking One (Nacalai Tesque, Kyoto, Japan). The membranes were then incubated overnight at 4°C with primary antibodies against GLI1 (sc-20687X, 1:5000 dilution; Santa Cruz Biotechnology), GLI2 (sc-28674, 1:1000 dilution; Santa Cruz Biotechnology), GLI3 (AF3690, 1:2000 dilution; R&D Systems, Minneapolis, MN, USA), and β-actin (PM053, 1:5000 dilution; MBL, Nagoya, Japan) in CanGet Signal Immunoreaction Enhancer Solution 1 (Toyobo Life Science, Osaka, Japan). After washing, secondary antibodies, peroxidase-conjugated anti-rabbit IgG (#7074; Cell Signaling Technology, Danvers, MA, USA) for GLI1, GLI2, and β-actin, or peroxidase-conjugated anti-goat IgG Light Chain Specific (205-032-176; Jackson ImmunoResearch, West Grove, PA, USA) for GLI3 were applied to the PVDF membranes at a dilution of 1:20,000 in CanGet Signal Immunoreaction Enhancer Solution 2 for 1 h at room temperature. Bound antibodies were detected using a chemiluminescence substrate (Western Lightning Ultra; PerkinElmer, Waltham, MA, USA) with a luminescent image analyzer (LAS 4000 Mini; GE Healthcare).

### Immunocytochemistry

The cells were fixed with 4% paraformaldehyde in PBS for 1 h. After washing, the nonspecific binding of antibodies was blocked with blocking buffer (Protein Block Serum-free; Dako, Glostrup, Denmark) at room temperature for 1 h. The cells were incubated with the anti-GLI1 antibody (AF3324, 1:40 dilution; R&D Systems), diluted at 1:1000 with Antibody Diluent (Dako), for overnight at 4°C. After washing several times in PBS with 0.05% Tween 20 (PBST, pH 7.6), the cells were incubated with Alexa Fluor 546-conjugated anti-goat antibodies (A11056, 1:200 dilution; Invitrogen) for 1 h in the dark. The cells were counterstained with DAPI/anti-fade mounting medium (Sigma, St. Louis, MO, USA). Images were captured with a BZ-X100(Keyence Inc. Osaka). We randomly observed 4 different areas in each condition and calculated the ratio of GLI1-positive cells to all cells in observed area.

### Alkaline phosphatase activity

The iPSCs were washed twice with ice-cold PBS and scraped in 10 mM Tris-HCl containing 2 mM MgCl_2_ and 0.05% Triton X-100, pH 9.8. After two cycles of freeze-thawing, the cell suspension was homogenized using a pellet pestle on ice. After centrifugation at 10 000 × g for 5 min at 4°C, a sample of each supernatant was transferred to tubes for the measurement of alkaline phosphatase (ALP) activity as the hydrolysis of ρ-nitrophenyl phosphate, using a LabAssay ALP kit (Wako Pure Chemicals Industries), and protein concentration, using a Protein Assay Rapid Kit (Wako Pure Chemicals Industries). The relative activity of each sample was reported as the ratio of its activity to the corresponding protein concentration.

### ALP staining

Following the osteogenic differentiation experiments, the iPSCs were washed twice with PBS, fixed in 4% paraformaldehyde for 10 min at room temperature, and washed twice more with distilled water. The fixed cells were stained with an ALP substrate solution (Roche Diagnostics, Basel, Switzerland) for 30 min at room temperature. The cells were then washed three times with distilled water, and images were captured using a phase-contrast microscope.

### RNA isolation and gene expression

Total RNA was extracted using QIAzol reagent (Qiagen Inc., Valencia, CA) according to the manufacturer’s instructions. cDNA was synthesized using a high-capacity cDNA reverse transcription kit (Applied Biosystems, Foster City, CA, USA).

The stemness and pluripotency of the iPSCs were analyzed using reverse transcription polymerase chain reaction (RT-PCR) analysis with ExTaq DNA polymerase (Takara Biotechnology, Shiga, Japan). The target genes analyzed included *OCT3/4*, *SOX2*, *c-MYC*, *KLF4*, *NANOG*, growth and differentiation factor 3 (*GDF3*), reduced expression 1 (*REX1*), fibroblast growth factor 4 (*FGF4*), α-fetoprotein (*AFP*), Msh homeobox 1 (*MSX1*), and microtubule-associated protein (*MAP*), with *β-actin* used as an internal control. The amplified PCR products were evaluated using electrophoresis on 2% agarose gels. The PCR primers are listed in Table 1.

**Table 1:** RT-PCR primers.

Gene symbol	Forward primer sequence	Reverse primer sequence
OCT3/4	gacagggggaggggaggagctagg	cttccctccaaccagttgccccaaac
SOX2	gggaaatgggaggggtgcaaaagagg	ttgcgtgagtgtggatgggattggtg
NANOG	cagccccgattcttccaccagtccc	cggaagattcccagtcgggttcacc
REX1	cagatcctaaacagctcgcagaat	gcgtacgcaaattaaagtccaga
MYC	gcgtcctgggaagggagatccggagc	ttgaggggcatcgtcgcgggaggctg
KLF4	acgatcgtggccccggaaaaggacc	tgattgtagtgctttctggctgggctcc
AFP	gaatgctgcaaactgaccacgctggaac	tggcattcaagagggttttcagtctgga
MSX1	cgagaggaccccgtggatgcagag	ggcggccatcttcagcttctccag
MAP	caggtggcggacgtgtgaaaattgagagtg	cacgctggatctgcctggggactgtg
SeVdp	agaccctaagaggacgaagacaga	actcccatggcgtaactccatag
β-actin	gggaaatcgtgcgtgacatta	ggcagtgatctccttctgcat

Quantitative real-time RT-PCR (qRT-PCR) analysis of GLI family zinc finger 1 (*GLI1*) and Runt-related transcription factor 2 *(RUNX2)* was conducted using Premix Ex Taq reagent (Takara Bio Inc., Shiga, Japan), according to the manufacturer’s instructions. *GLI1* and *RUNX2* levels levels were normalized to those of *GAPDH*. The primers and probes used in the qRT-PCR assays are listed in Table 2. Relative expression levels of the target genes in patient-derived iPSCs were calculated as the fold-change in expression levels relative to the corresponding gene in the control (KD) iPSCs (KDiPSCs).

**Table 2:** The primers used in the qRT-PCR assays.

Gene symbol	Forward primer sequence	Revers primer sequence
GLI1	ccagccagagagaccaacag	cccgcttcttggtcaactt
RUNX2	gtgcctaggcgcatttca	gctcttcttactgagagtggaagg
GAPDH	cggacaggattgacagattg	cgctccaccaactaagaacg

The RT^2^ Profiler PCR Array (Human Hedgehog Signaling Pathway No. 330231 PAHS-078ZA, Qiagen) was used to assess the expression patterns of components of the Hh signaling pathway following osteogenic induction. We used RT^2^ Profiler PCR Arrays. A single plate was applied for each case. The expression level of each target gene was calculated using the ΔΔC_t_ method. Expression levels of the target genes were normalized to those of housekeeping genes, according to the manufacturer’s recommendations (http://www.sabiosciences.com/dataanalysis.php). The fold-change is calculated by the equation 2(-ΔΔC_t_). For the fold-regulation, the software transforms fold-change values less than 1 (meaning that the gene is down regulated) by returning the negative inverse. Gene expression levels in patient-derived iPSCs were expressed as the fold-regulation relative to the expression levels in KDiPSCs.

### Statistical analysis

The data are expressed as the mean ± standard deviation (SD). When an analysis of variance test detected differences among experimental groups, multiple comparisons between each group were conducted using Bonferroni testing. Statistical significance was defined as *p*< 0.05.

## Results

### Clinical features of the Gorlin syndrome patients

The clinical features of the four Gorlin syndrome patients enrolled in this study are summarized in Table 3. The diagnosis of Gorlin syndrome was based on the six major and six minor disease criteria described by Kimonis *et al* [23] (S1 Table). All four patients met at least two of the major criteria, and *PTCH1* mutations were identified in all four [17].

**Table 3:** Clinical features of Gorlin syndrome patients.

	Patient information		Clinical features							
			Major criteria						Minor criteria	Other
Sample	Birth year	Sex	>2 BCCs, or 1 BCC in patients <20 years old	Odontogenic keratocysts of the jaw*	Three or more palmar or plantar pits	Bilamellar calcification of the falx cerebri	Bifid, fused, or markedly splayed ribs	First-degree relative with Gorlin syndrome	Congenital malformations	
Gorlin1	1953	M	Yes	Yes	Yes		Yes	Yes	Hypertelorism	
Gorlin2	1987	M	Yes	Yes	Yes			Yes		Hydrocephaly
Gorlin3	1983	M		Yes	Yes	Yes			Hypertelorism	
Gorlin4	1995	M		Yes			Yes		Hypertelorism	Mental retardation, autism

*As determined by histological analysis.

### Generation and characterization of iPSCs derived from the Gorlin syndrome patients

**Fig 1:**
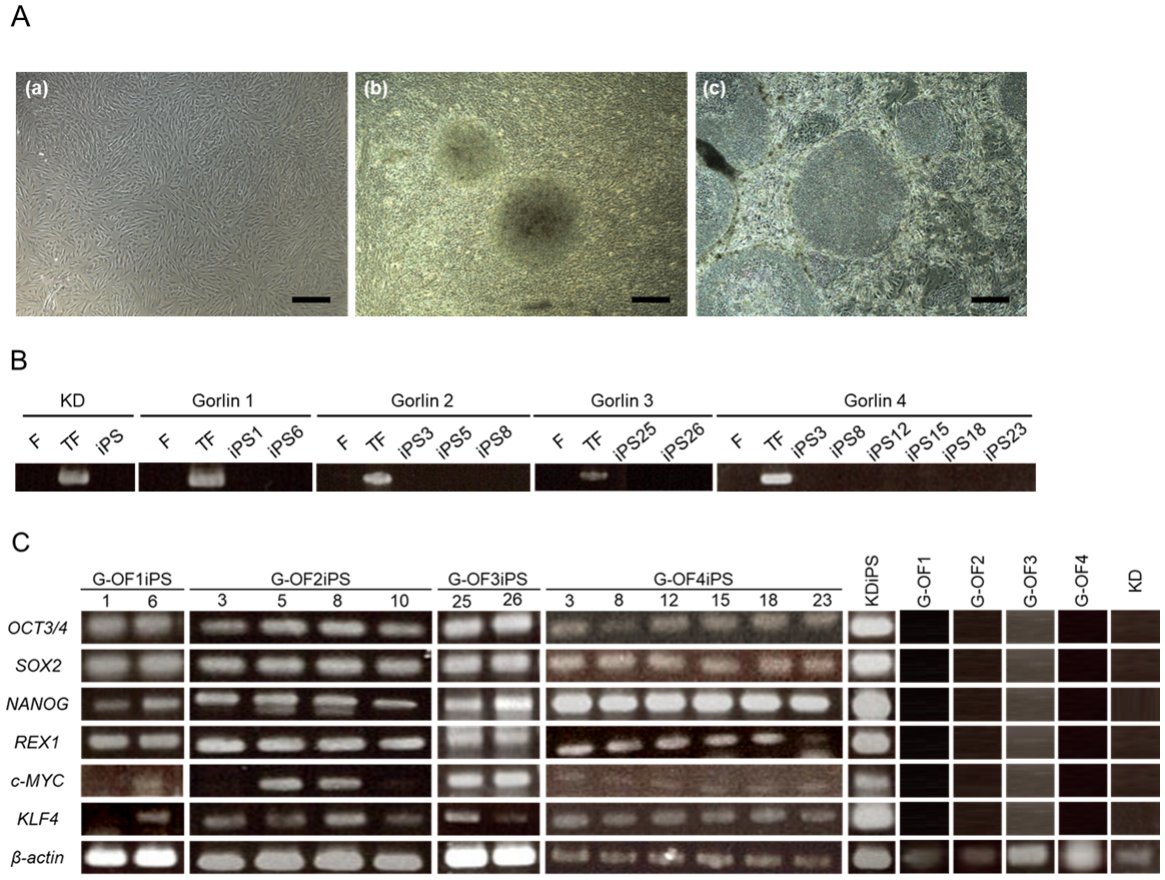
Generation of iPSCs from Gorlin syndrome fibroblasts (G-OFs). (A) We collected fibroblasts from surgically resected unaffected oral skin areas. Typical spindle shape fibroblasts were obtained (a). Fibroblasts (1 × 10^5^/well) were transfected with the Sendai virus vector SeVdp(KOSM)302L. The resulting iPSC colonies were selected 21–25 days after transfection and several clones were subsequently expanded (b). Stable clones (two clones of Patient 1, four clones of Patient 2, two clones of Patient 3, and six clones of Patient 4) exhibited the characteristic morphology of human iPSCs. Representative iPSCs (from passage number 6) are shown (c) (scale bar: 400 μm). (B) We confirmed the infection and silencing of SeVdp using RT-PCR. Cells expressed SeVdp mRNA one day after infection (TF). SeVdp was not detected in any G-OFs (F) or G-OFiPSCs. (C) RT-PCR analysis of embryonic stem cell marker genes in patient-derived iPSCs (G-OF1iPS clones 1 and 6; G-OF2iPS clones 3, 5, 8, and 10; G-OF3iPS clones 25 and 26; and G-OF4iPS clones 3, 8, 12, 15, 18, and 23) and control iPSCs (KDiPSCs). *β-actin* was used as a loading control. The target genes evaluated included *OCT3/4*, *SOX2*, *NANOG*, *REX1*, *c-MYC*, and *KLF4*.

Cultured patient fibroblasts (G-OFs; Fig 1A(a)) were preprogrammed by infection with the Sendai virus vector SeVdp(KOSM)302L. Cells with an embryonic stem cell-like morphology were first detectable 15 or 16 days after infection (Fig 1A(b)). The colonies were collected 21–25 days after infection and transferred to 24-well plates with SNL76/7 feeder cells in primate embryonic stem cell medium supplemented with bFGF (Fig 1A(c)). SeVdp infection and silencing were confirmed using RT-PCR (Fig 1B). We also confirmed that all the patient-derived iPSC lines expressed the embryonic stem cell markers *OCT3/4*, *SOX2*, *NANOG*, *REX1*, *MYC*, and *KLF4* (Fig 1C) and were capable of forming teratomas. A previous study demonstrated that RNA encoded by the Sendai vector can be silenced using siRNA [20]; from this, we concluded that the marker genes were endogenously expressed by the iPSCs.

**Fig 2:**
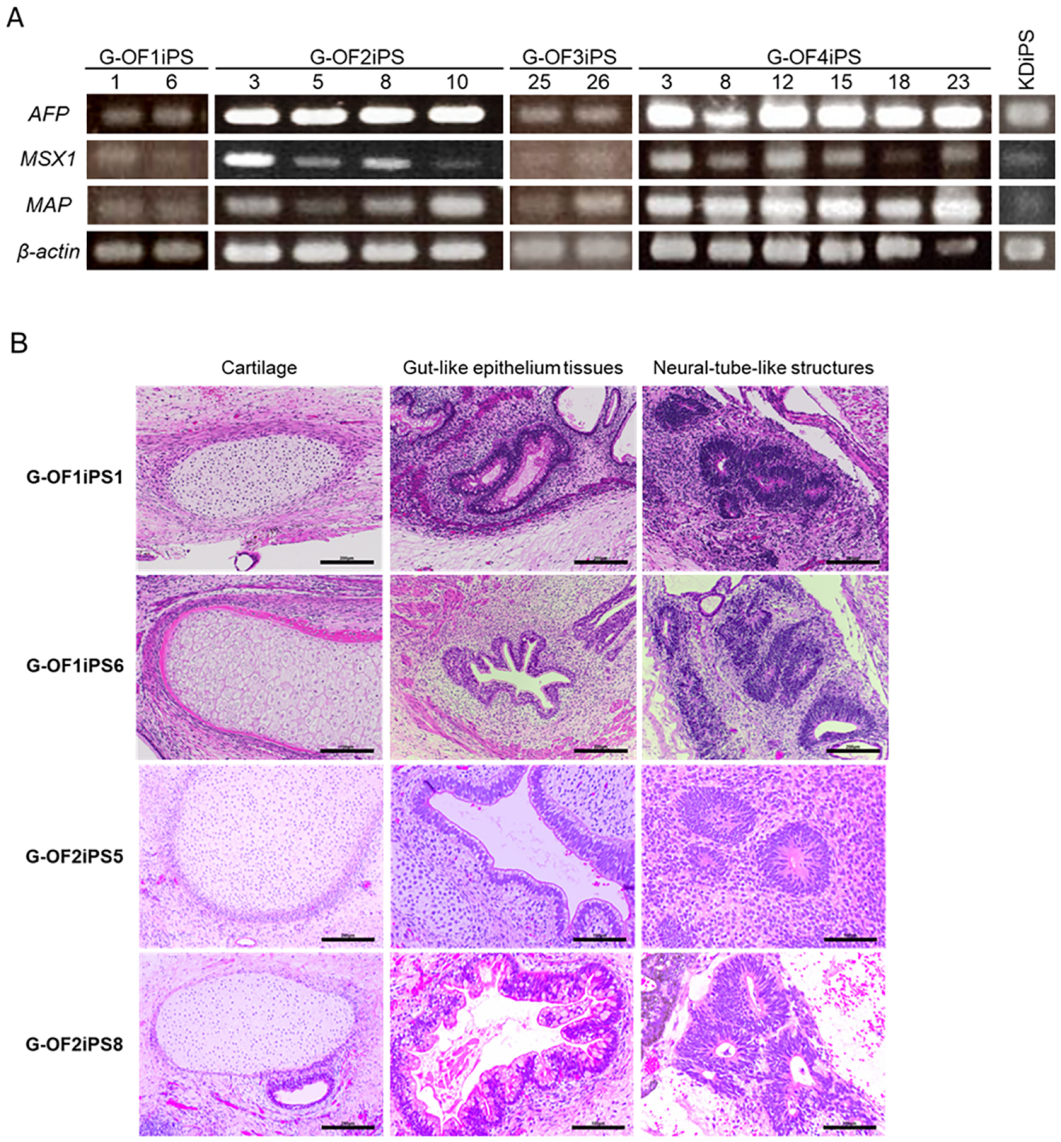
Pluripotency of patient-derived iPSCs. (A) Embryoid bodies expressed differentiation markers specific to all three germ layers. To examine the differentiation potential of iPSCs, we evaluated their ability to form embryoid bodies by using floating culture assays. RT-PCR was used to examine the expression of differentiation markers in the embryoid bodies. *AFP* was used as an endodermal marker, *MSX1* as a mesodermal marker, and *MAP* as an ectodermal marker, with *β-actin* used as an internal control. (B) Teratoma formation in G-OFiPSCs. Patient-derived iPSCs (G-OF1iPS clones 1 and 6, and G-OF2iPS clones 5 and 8) were injected at a concentration of 1 × 10^6^ cells in 20 μl of PBS into 8–10-week-old male CB17 SCID mice. Tumors were collected 9–12 weeks after the injections. Histological examination demonstrated that tissues originating from all three embryonic germ layers were present, including cartilage (mesoderm), gut-like epithelium (endoderm), and neural tube-like structures (ectoderm).

EB formation was observed after 6–10 days in floating culture. The EBs maintained in floating culture expressed markers of all three germ layers: *AFP* (endoderm), *MSX1* (mesoderm), and *MAP* (ectoderm) (Fig 2A). Teratoma formation was assessed by injecting 1 × 10^6^ SeVdp-iPS cells (G-OF1iPS1, G-OF1iPS16, G-OF2iPS5, or G-OF2iPS8) into the testes of SCID mice. Tumors were recovered 9–12 weeks after the injections and subsequently fixed, paraffin-embedded, and sectioned (4 μm) for hematoxylin and eosin staining. This identified tissues representing all three embryonic germ layers (Fig 2B), indicating that the cells were pluripotent.

### Activation of the Hh pathway in patient-derived iPSCs

**Fig 3:**
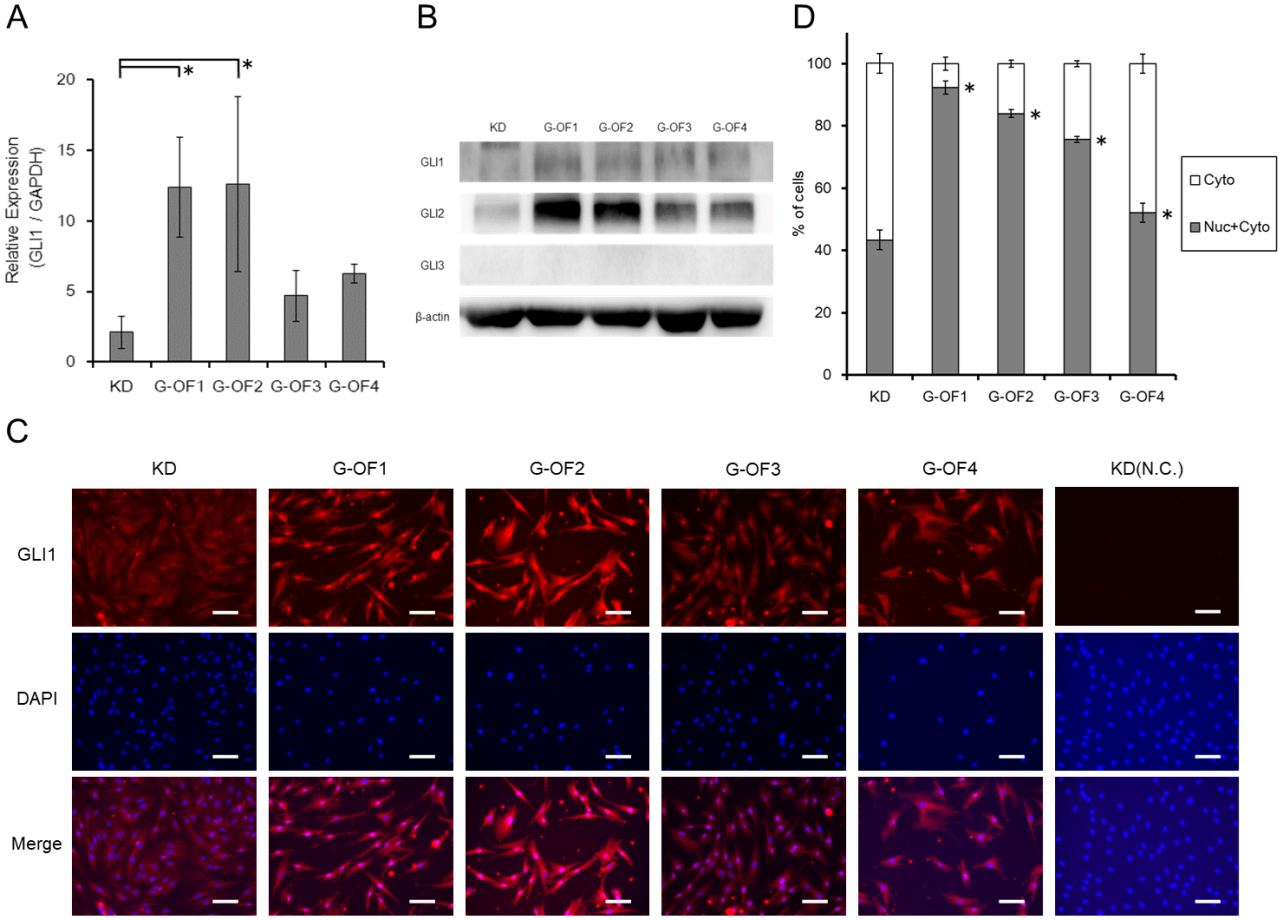
Activation of Hh signaling in patient-derived fibroblasts and iPSCs. (A) GLI1 expression in fibroblasts under normal culture conditions. Under normal culture conditions, mean GLI1 mRNA levels were approximately 2-fold greater in G-OFs than in control KD fibroblasts. (B)Protein expression of endogenous GL1,GLI2 under normal culture conditions. (C)Intracellular expression was confirmed by GLI1 staining(red). DAPI staining(blue) delineates the nuclear (scale bar: 100μm). (D)The proportion of cells staining GLI1 was calculated among all the cells stained with DAPI. A high rate of the nuclear translocation of GLI1 was showed in G-OFs. Statistical significance comparered to KD fibroblast was calculated. Values represent the mean ± SD. Bonferroni correction for multiple comparisons was applied, *p < 0.05.

**Fig 4:**
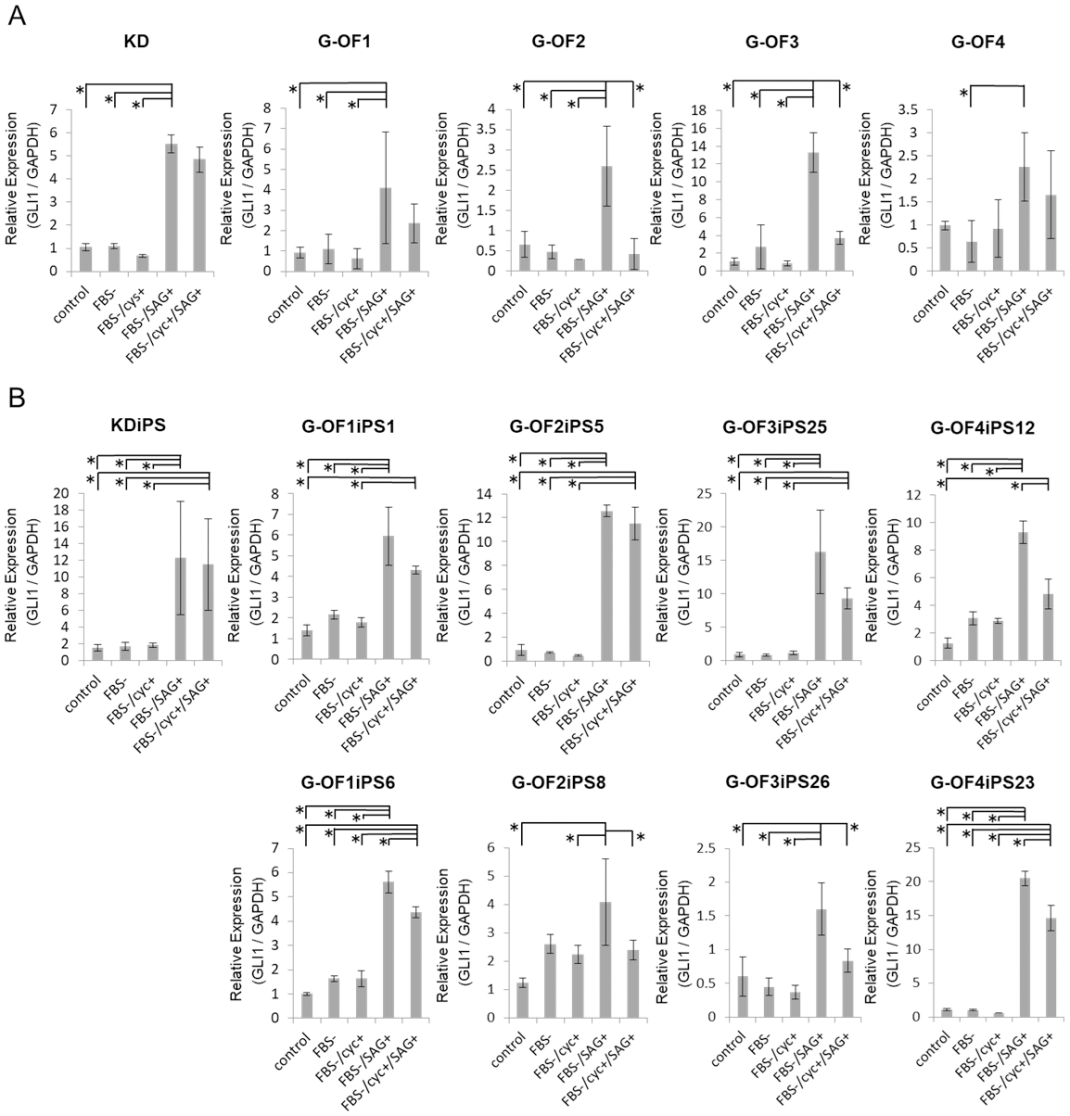
Expression of GLI1 in serum starved culture. (A) GLI1 expression in fibroblasts grown under serum starvation conditions (DMEM with 0.5% or 0% FBS). The SMO agonist SAG (1 μM) enhanced GLI1 expression levels, and this effect was reversed by the SMO antagonist cyc (100 nM). (B) Control and patient-derived iPSCs (KDiPSCs and G-OFiPSCs, respectively) treated as in (E). SAG enhanced GLI1 expression levels, and this effect was reversed by cyc. Values represent the mean ± SD. Bonferroni correction for multiple comparisons was applied, *p < 0.05.

We observed a <2-fold increase in mean *GLI1* expression levels in G-OFs compared with that in control human fibroblasts (Fig 3A) cultured in serum-containing medium. In both control and patient-derived fibroblasts cultured under serum starvation conditions, the SMO agonist SAG enhanced *GLI1* expression (Fig 3A). We examined the protein expression of *GLI1*, *GLI2*, and *GLI3* as target molecules of the Hedgehog pathway. As shown in Fig 3A 3B and 3D, *GLI1* protein expression in Gorlin fibroblasts increased significantly. We also examined Gli localization in Gorlin fibroblasts by immunohistochemistry, finding increased immunopositivity in every Gorlin fibroblast with more than 50%–90% of the *GLI1*-positive cells having *GLI1*-positive nuclei (Fig 3C and 3D). To confirm the upregulation of Hh pathway, we investigated whether the SMO inhibitor cyclopamine reversed this effect (Fig 4A and 4B). Similarly, SAG upregulated *GLI1* expression in both patient-derived iPSCs (G-OFiPSCs) and control iPSCs (KDiPSCs) grown under serum starvation conditions (Fig 4A and 4B). These observations suggest that serum starvation conferred hypersensitivity to Hh signaling activity.

**Fig 5:**
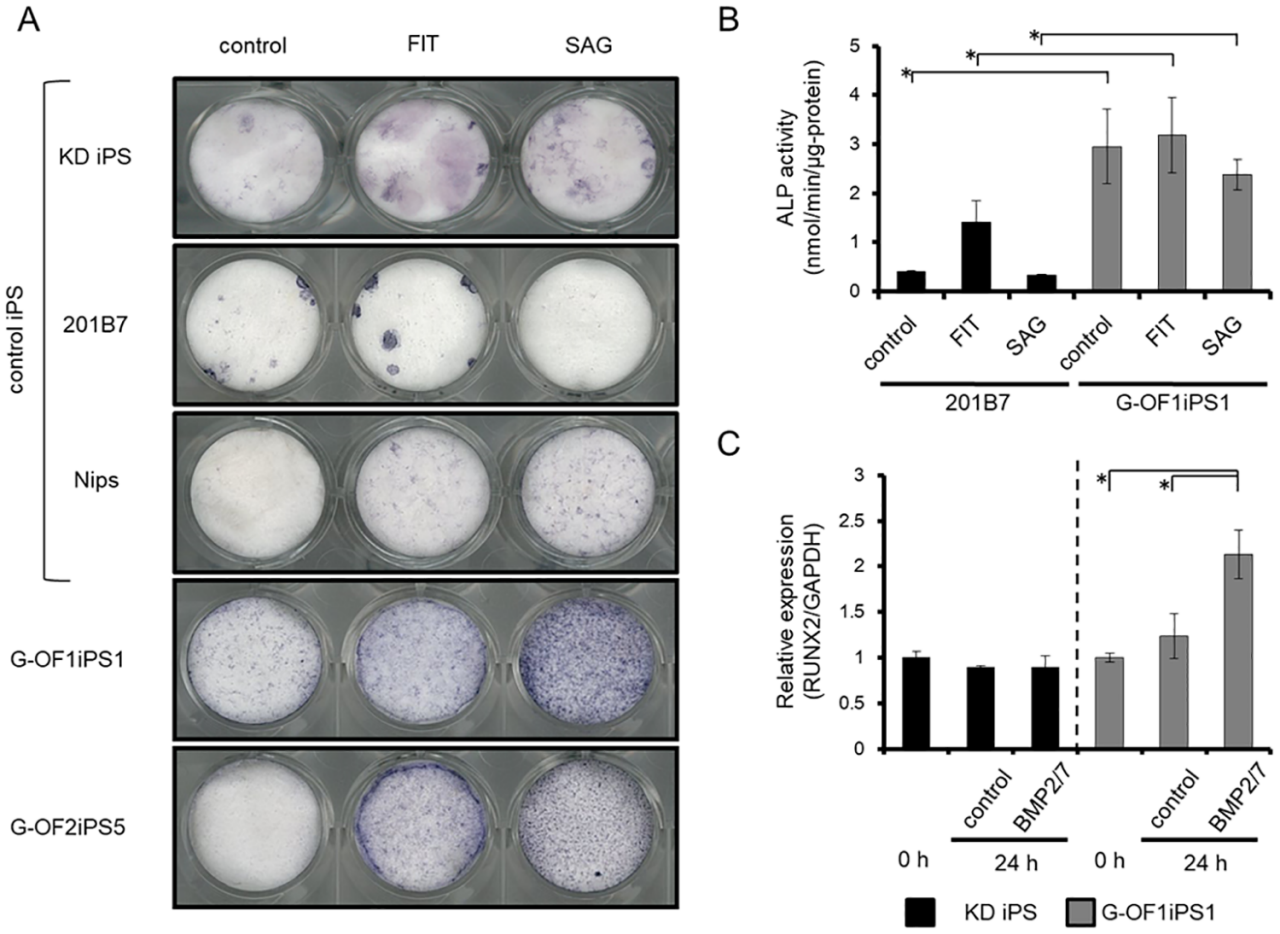
Hh-dependent induction of osteogenic marker ALP in patient-derived iPSCs. (A) Control iPSCs (KD iPSCs, 201B7iPS cells, and Nips B2 iPSCs) and patient-derived iPSCs (G-OF1iPS1, and G-OF2iPS5) were cultured in OBM, OBM with FIT (bFGF, IGF-1, and TGF-β1), or OBM with SAG. FIT- and SAG-induced ALP activity significantly increased in patient-derived iPSCs compared with control iPSCs. (B) A sample of each supernatant was transferred to tubes for measurement of ALP activity as the hydrolysis of ρ-nitrophenyl phosphate using a LabAssay ALP kit and protein concentration using a Protein assay rapid kit. G-OF1iPS1 was showed a high ALP activity compared with 201B7,especially cultured in OBM with SAG. (C) KDiPSCs and G-OF1iPS1 cells were cultured in OBM (control), or OBM supplemented with 100 ng/ml BMP2/7 for 24 h. qRT-PCR analysis of RUNX2 was conducted using Premix Ex Taq reagent according to the manufacturer’s instructions. RUNX2 levels were normalized to those of GAPDH. A significant difference was observed in G-OF1iPS1. Especially, BMP2/7-induced RUNX2 expression promoted an approximate 2-fold increase in the number of G-OFiPSCs compared with KDiPSCs. Values represent the mean ± SD. Bonferroni correction for multiple comparisons was applied, *p < 0.05.

Next, we investigated osteogenesis in iPSCs using FIT (a combination of bFGF, IGF-1, and TGF-β) with or without SAG, as described in our recent report [21]. FIT, but not SAG, induced the activity of ALP, a marker of osteogenic induction, in three different control iPSC lines as well as in patient-derived iPSCs (Fig 5A). By contrast, SAG significantly enhanced ALP staining only in patient-derived iPSCs. We performed an ALP assay and observed results consistent with the ALP staining results (Fig 5B). We have previously reported that bone morphogenetic protein (BMP), which induces ectopic bone formation, failed to induce ALP expression during osteogenesis in human iPSCs [21]. Consistent with this finding, treatment with BMP2 and BMP7 (BMP2/7) did not enhance the expression of *RUNX2* in control iPSCs. By contrast, BMP2/7 significantly enhanced *RUNX2* expression levels in G-OFiPSCs (Fig 5C). These results suggest that, in comparison with control iPSCs, G-OFiPSCs are hypersensitive to Hh-induced osteogenesis.

### Differential expression of Hh pathway- and osteogenesis-associated genes in patient-derived iPSCs

**Fig 6:**
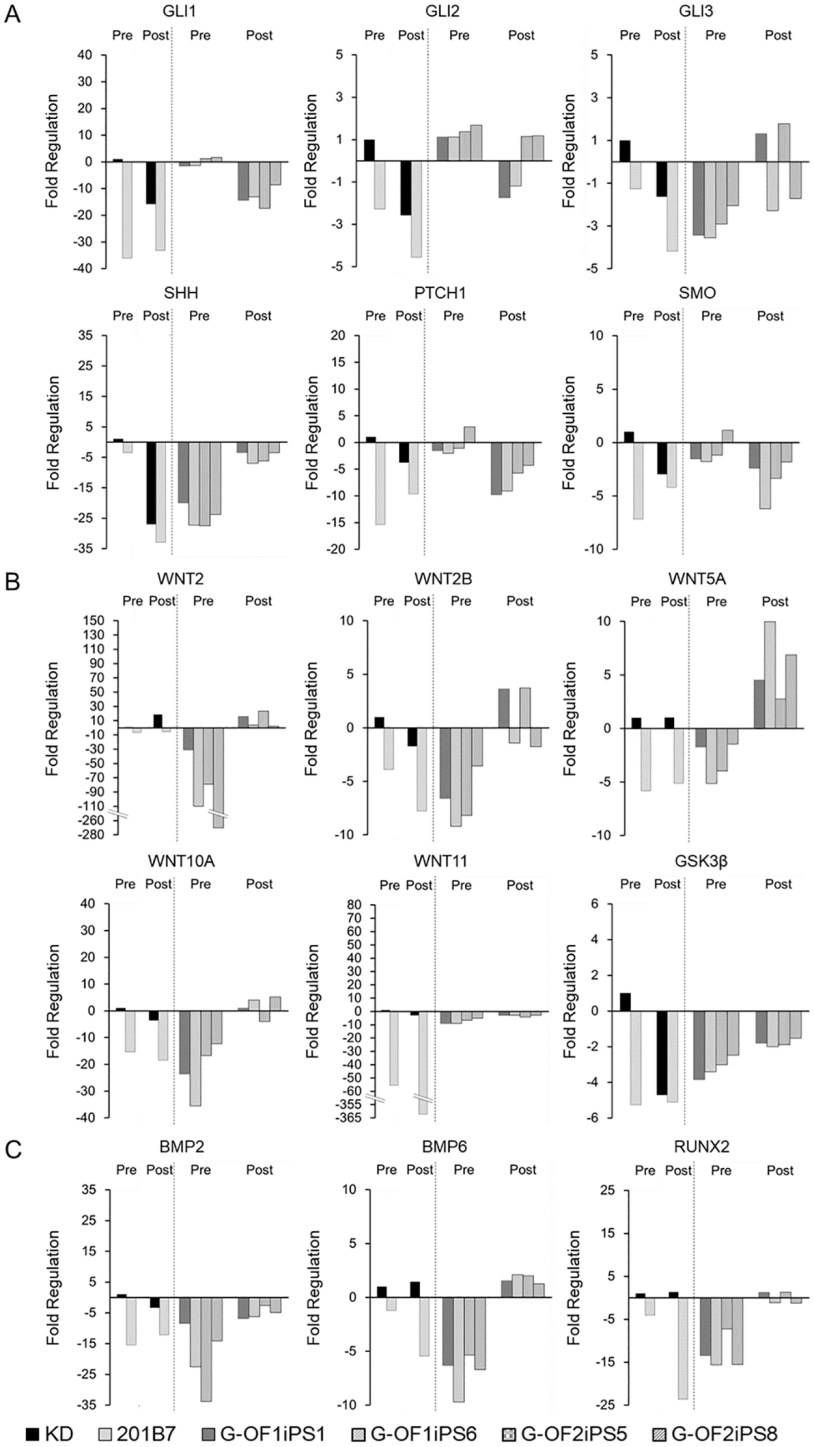
Distinct expression patterns of Hh-associated genes in control and patient-derived iPSCs under osteogenic conditions. We investigated two controls (KDiPS and 201B7) and four Gorlin iPS cases derived from different patients. Because we used single RT^2^ Profiler PCR Array plate for each case, we did not performed statistical analysis. (A) Osteogeic induction promoted characteristic changes in the expression levels of the following genes associated with the canonical Hh pathway: *GLI1*, *GLI2*, *GLI3*, and *SHH*. Compared with the basal conditions, *GLI1* and *GLI2* expression levels decreased in both controls (KDiPSCs and 201B7) and G-OFiPSCs grown in OBM, whereas *GLI3* expression increased only in G-OFiPSCs grown in OBM. Basal expression levels of SHH was significantly lower in G-OFiPSCs compared with controls (KDiPSCs and 201B7). However, OBM enhanced *SHH* expression levels in G-OFiPSCs but not in control cells. (B) The basal expression levels of many Wnt genes were lower in patient-derived iPSCs than in control iPSCs. With the exception of *WNT2*, all of the Wnt genes evaluated were upregulated in G-OFiPSCs grown in OBM and downregulated in control cells (KDiPSCs and 201B7) grown in OBM. (C) Changes in *BMP2*, *BMP6*, and *RUNX2* expression levels were greater in patient-derived iPSCs than in controls (KD iPSCs and 201B7). The ΔΔC_t_ value 10 days after OBM treatment (Post) was determined, and the fold-change relative to pre-induction (Pre) KDiPSCs was calculated for each gene.

To determine whether the enhanced response of patient-derived iPSCs to osteogenic induction was directly mediated by Hh signaling, we analyzed the expression levels of 84 Hh- and osteogenesis-associated genes using a RT-PCR array. We observed substantial variability in the expression levels of multiple Hh pathway-associated genes, including *PTCH1*, *GLI1*, *GLI2*, *GLI3*, *SMO*, and *SHH*, and in osteogenesis-related genes in cell lines grown under normal culture conditions (basal expression) and in cells grown in OBM (S2 Table). As shown in Fig 6A, basal expression levels of *SHH* decreased in all G-OFiPSC lines compared with the levels in the control iPSCs. However, *SHH* was upregulated in G-OFiPSCs grown in OBM and downregulated in control iPSCs (KDiPSCs and 201B7) grown in OBM. The expression of *GLI1* and *GLI2*, which are both activators of gene expression, substantially decreased in both control iPSCs (KDiPSCs and 201B7) and patient-derived iPSCs grown in OBM. By contrast, *GLI3*, which functions as a transcriptional repressor, was upregulated in patient-derived iPSCs and downregulated in the control iPSCs (KDiPSCs and 201B7) grown in OBM, similar to the findings for *SHH* (Fig 6A). This differential reaction of the target molecules could account for the enhanced response of the Hh pathway to osteogenic induction.

Hh proteins, Wnt proteins, and BMPs secreted during embryonic development all contribute to cell fate determination. The Wnt proteins comprise a large family of protein ligands that affect diverse cellular processes, and recent studies have indicated that Wnt signaling plays a key role in the maintenance of stem cell pluripotency. The G-OFiPSCs tended to express lower basal levels of most Wnt genes, including *WNT2*, *2B* (formerly *WNT13*), *5A*, *10A*, and *11*, than the control iPSCs (KDiPSCs and 201B7) (Fig 6B and S2 Table). Wnt signaling is also critical for the control of osteoblastogenesis and bone formation. The expression levels of several Wnt genes, including *WNT2*, *2B*, *5A*, *5B*, *6*, *10A*, and *11*, increased following osteogenic induction in G-OFiPSCs, whereas the expression levels of these genes either decreased or remained unchanged in the control iPSCs (KDiPSCs and 201B7). BMPs and RUNX2 are also important osteogenic differentiation factors, and RUNX2 is regarded as the master regulator of osteogenesis. As shown in Fig 6C, *BMP2*, *BMP6*, and *RUNX2* were strongly upregulated by OBM in G-OFiPSCs but not in control KDiPSCs and 201B7 cells. These reaction trends were also observed in the expression of other BMP molecules (S1 Fig). Together, these findings demonstrated that the expression levels of osteogenic-inducing Hh, Wnt, and BMP genes, as well as *RUNX2*, were upregulated in G-OFiPSCs but not in control iPSs during osteogenic induction, suggesting that G-OFiPSCs were primed to a greater extent for osteogenic differentiation compared with the control iPSCs.

## Discussion

This study resulted in three major findings. First, we generated iPSCs from OFs derived from four unrelated Gorlin syndrome patients. Second, we demonstrated that patient-derived iPSCs (G-OFiPSCs) were hypersensitive to osteogenic inducers: ALP activity induced by FIT, SAG, and FIT + SAG increased in patient-derived iPSCs compared with control iPSCs. Furthermore, the Hh receptor agonist SAG substantially affected osteogenic induction in patient-derived iPSCs but not in control iPSCs. This effect was potentially due to the weaker suppressive effects of mutant PTCH1 on SMO [17,18,24–27]. Third, basal levels of *SHH*, *IHH*, the Hh pathway modulator *HHAT*, and several *Wnt* and *BMP* genes were lower in G-OFiPSCs than in control cells, whereas these genes were expressed at higher levels in G-OFiPSCs under osteogenesis-inducing conditions. Collectively, these results suggest that the skeletal abnormalities characteristic of Gorlin syndrome could be due to stem cell hypersensitivity to osteogenic induction resulting from constitutive Hh signaling.

In the unstimulated state, PTCH1 represses SMO. The binding of PTCH1 to Hh proteins promotes the release of SMO and the subsequent activation of downstream targets. It has been predicted that heterozygous germline loss-of-function *PTCH1* mutations would inhibit PTCH1-mediated SMO suppression, thereby promoting the upregulation of Hh-mediated pathways [28,29]. Therefore, loss-of-function *PTCH1* mutations may lead to constitutively active Hh signaling [2–8,30–32]. *GLI1* expression levels significantly increased in patient-derived fibroblasts and iPSCs compared with in the corresponding control cells. A previous study demonstrated that serum starvation in combination with SHH treatment synergistically enhanced *GLI1* expression in G-OFs [33]. We found that *GLI1* expression was enhanced in all of the cells examined under serum starvation conditions in the presence or absence of SAG, indicating that serum starvation is a universal trigger of Hh signaling activation *in vitro*. However, because serum starvation conditions cannot be recapitulated *in vivo*, we examined under normal serum conditions osteogenesis in response to an established combination of osteogenic cytokines (FIT) [21]. Under these conditions, osteogenesis was enhanced in patient-derived iPSCs but not in control iPSCs. Similarly, SMO activation alone induced osteogenesis in patient-derived iPSCs but not in control iPSCs, suggesting that G-OFiPSCs are hypersensitive to Hh-mediated osteogenesis. This hypothesis is consistent with the findings of previous reports that both Gorlin syndrome patients with heterozygous loss-of-function *PTCH1* mutations and *Ptch1*-deficient (*Ptch1*^+/−^) mice exhibited increased bone mass as adults and that osteoblast differentiation was accelerated in *Ptch1*^+/−^ mouse cells [9].

The activation of Hh receptors triggers the nuclear localization of GLI transcription factors, which in turn drive the expression of the Hh target genes involved in osteogenesis as well as cell proliferation, survival, angiogenesis, and the epithelial-to-mesenchymal transition. It is therefore reasonable to hypothesize that aberrant Hh signaling in Gorlin syndrome patients might contribute to their susceptibility to tumor formation [2,23,34–40]. Basal expression levels of *SHH*, *IHH*, and *HHAT* were significantly lower in G-OFiPSCs than in control iPSCs, whereas basal expression levels of *SMO*, *GLI1*, and *GLI2* were greater in the patient-derived iPSCs. This expression pattern further supports the hypothesis that Hh signaling is constitutively active in G-OFiPSCs. The downregulation of *IHH* and *SHH* in G-OFiPSCs could be a consequence of a negative feedback loop triggered by constitutive Hh signaling. Small molecule inhibitors of HHAT have been investigated as potential anti-cancer agents [41,42]. Thus, the downregulation of *HHAT* in patient-derived iPSCs might also contribute to tumorigenesis in patients with Gorlin syndrome.

We observed that basal expression levels of genes associated with the Wnt pathway were substantially lower in patient-derived iPSCs. Coordinated regulation of the Hh and Wnt pathways is observed in many cancers [3,4,32]. In colorectal cancer, *GLI1* overexpression inhibits Wnt signaling and colorectal cancer cell proliferation, even in cells that harbor a stabilizing mutation in *β-catenin* [43–45]. During neural development, elevated Hh signaling activity suppresses the canonical Wnt pathway in the cranial nerve region [46]. Thus, constitutively active Hh activity may account for the lower basal expression levels of Wnt-associated genes observed in Gorlin syndrome cells. However, Hh signaling promotes the expression of multiple Wnt genes in G-OFiPSCs under osteogenic conditions.

BMPs are well-characterized osteogenic factors. We found that *BMP4* and *BMP6* levels markedly increased in G-OFiPSCs following osteogenic induction. We previously reported that BMP2/7 did not induce osteogenesis in human iPSCs [21]. In the present study, BMP2/7 significantly upregulated the BMP target gene and master regulator of osteogenesis *RUNX2* in patient-derived iPSCs cultured in OBM but not in control iPSCs cultured in OBM. We propose that osteogenic conditions may contribute to calcification in the falx cerebri in Gorlin syndrome patients.

We also observed that *FOXE1* expression decreased in patient-derived iPSCs compared with the control iPSCs. FOXE1 is a transcription factor that regulates craniofacial morphogenesis [47,48]. Therefore, the downregulation of *FOXE1* may be related to the craniofacial abnormalities, such as mild microcephaly, observed in some Gorlin syndrome patients [2,23,27].

In conclusion, Hh signaling activity is enhanced in iPSCs generated from Gorlin syndrome patients. Furthermore, these patient-derived iPSCs were hypersensitive to the Hh activator SAG and other osteogenic inducers, even when grown in media with a high serum concentration. Thus, Gorlin syndrome patient-derived iPSCs are highly responsive to osteogenic differentiation induced by the overexpression of Hh genes and genes that encode various Hh-associated signaling molecules, including Wnts, BMPs, and RUNX2. Gorlin syndrome patient-derived iPSCs could be a useful tool not only for investigating the pathogenesis of Gorlin syndrome and developing new treatments but also for cancer research more widely.

## Supporting information

S1 Fig Expression patterns of Hh-associated genes under osteogenic conditions. (TIF)

S1 Table Gorlin syndrome diagnostic criteria. (DOCX)

S2 Table Changes in gene expression in patient-derived iPSCs and control iPSCs (KDiPSCs) induced by OBM. (DOCX)
