# Supplementary figures and images for: Gorlin syndrome-derived induced pluripotent stem cells are hypersensitive to hedgehog-mediated osteogenic induction
Source: PLoS One. 2017 Oct 31;12(10):e0186879. doi: 10.1371/journal.pone.0186879 (PMC5663396; doi:10.1371/journal.pone.0186879)

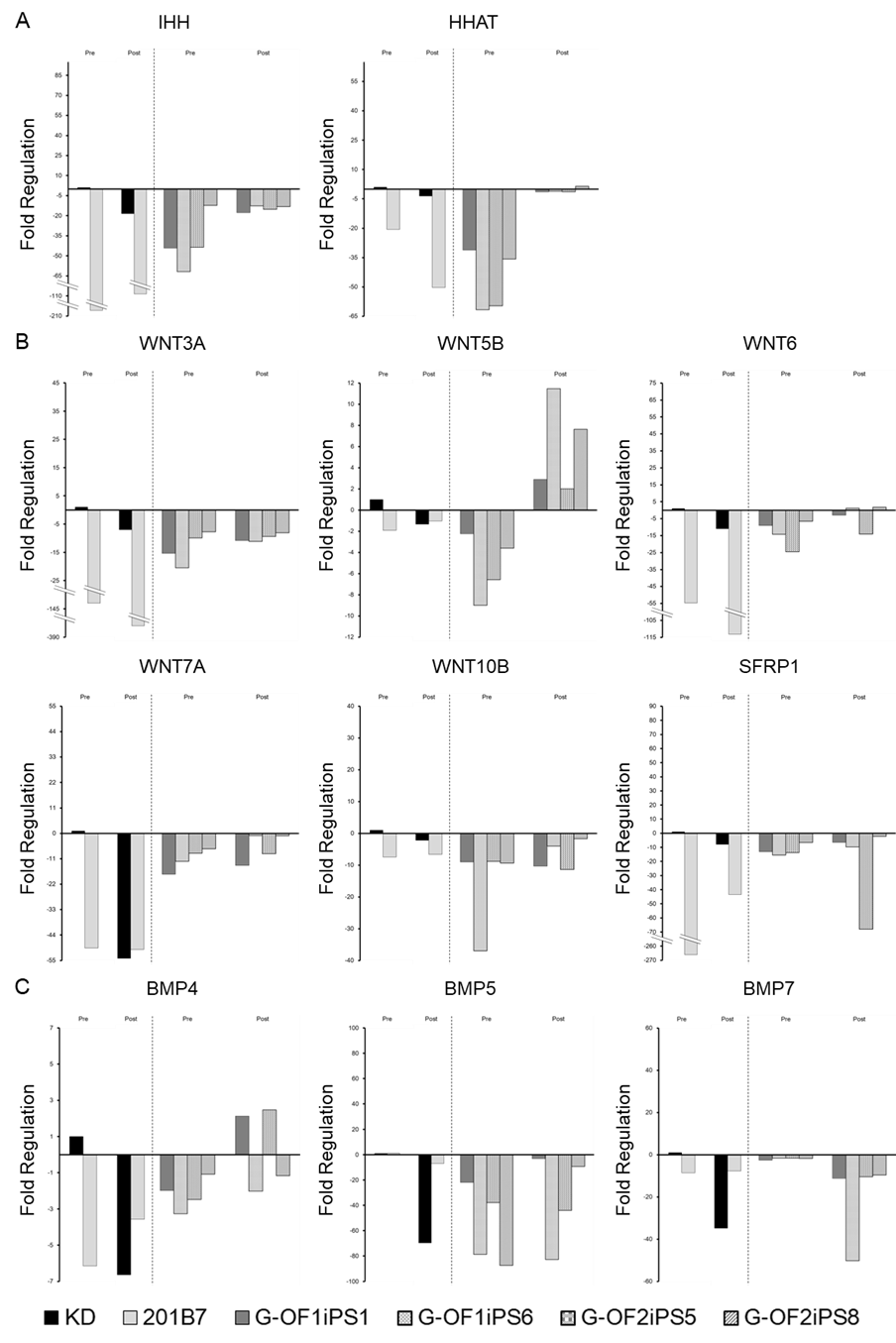

Supplement: S1 Fig — (TIF) [file pone.0186879.s001.tif]
