# Supplementary material for: Gorlin syndrome-derived induced pluripotent stem cells are hypersensitive to hedgehog-mediated osteogenic induction
Source: PLoS One. 2017 Oct 31;12(10):e0186879. doi: 10.1371/journal.pone.0186879 (PMC5663396; doi:10.1371/journal.pone.0186879)
Supplement: S1 Table — (DOCX) [file pone.0186879.s002.docx]

| A diagnosis of Gorlin syndrome is based on the presence of two major criteria, or one major and two minor criteria. |
| --- |
| Major criteria |
| 1. More than two BCCs, or one BCC in patients <20 years old |
| 2. Odontogenic keratocysts of the jaw (based on histology) |
| 3. Three or more palmar or plantar pits |
| 4. Bilamellar calcification of the falx cerebri |
| 5. Bifid, fused, or markedly splayed ribs |
| 6. A first-degree relative with Gorlin syndrome |
| Minor criteria |
| 1. Macrocephaly (after adjustment for height) |
| 2. Congenital malformations: cleft lip or palate, frontal bossing, “coarse face,”  moderate or severe hypertelorism |
| 3. Other skeletal abnormalities: Sprengel deformity, marked pectus deformity,  marked syndactyly of the digits |
| 4. Radiological abnormalities: Bridging of the sella turcica, vertebral anomalies such as  hemivertebrae, fusion, or elongation of the vertebral bodies, modeling defects of  the hands and feet, flame-shaped lucencies of the hands or feet |
| 5. Ovarian fibroma |
| 6. Medulloblastoma |
