# Supplementary material for: Gorlin syndrome-derived induced pluripotent stem cells are hypersensitive to hedgehog-mediated osteogenic induction
Source: PLoS One. 2017 Oct 31;12(10):e0186879. doi: 10.1371/journal.pone.0186879 (PMC5663396; doi:10.1371/journal.pone.0186879)
Supplement: S2 Table — (DOCX) [file pone.0186879.s003.docx]

|  | Log10 (Fold-change) | | | | | | | | | |
| --- | --- | --- | --- | --- | --- | --- | --- | --- | --- | --- |
|  | KDiPS (control) | KDiPS OBM | G-OF1 iPS1 | G-OF1iPS1 OBM | G-OF1iPS6 | G-OF1iPS6 OBM | G-OF2iPS5 | G-OF2iPS5 OBM | G-OF2iPS8 | G-OF2iPS8 OBM |
| *SHH* | 0 | −1.25964 | −0.84103 | −0.25885 | −0.96138 | −0.5099 | −1.0278 | −0.45942 | −1.07058 | −0.27515 |
| *IHH* | 0 | −1.0942 | −1.18709 | −0.97551 | −1.31695 | −0.77186 | −1.22915 | −0.85078 | −0.78436 | −0.85605 |
| *HHAT* | 0 | −0.38352 | −1.03386 | 0.135451 | −1.31695 | 0.293804 | −1.36452 | 0.184833 | −1.24795 | 0.402175 |
| *PTCH1* | 0 | −0.40461 | 0.266702 | −0.7164−7 | 0.167376 | −0.62434 | 0.38412 | −0.4232 | 0.76883 | −0.36241 |
| *GLI1* | 0 | −1.0278 | 0.260691 | −0.88506 | 0.347993 | −0.78383 | 0.510545 | −0.90483 | 0.524993 | −0.66354 |
| *GLI2* | 0 | −0.23905 | 0.510545 | 0.033102 | 0.525602 | 0.257679 | 0.549677 | 0.398565 | 0.531019 | 0.338954 |
| *GLI3* | 0 | 0.04034 | −0.07645 | 0.394347 | −0.07645 | −0.0253 | −0.05237 | 0.585201 | −0.0048 | 0.031893 |
| *SMO* | 0 | −0.29921 | 0.275749 | −0.10535 | 0.224559 | −0.4588 | 0.335959 | −0.18842 | 0.365451 | 0.004837 |
| *WNT2* | 0 | 1.437719 | −1.03058 | 1.472036 | −1.56703 | 0.931986 | −1.48545 | 1.705034 | −2.12494 | 0.618916 |
| *WNT2B* | 0 | −0.05839 | −0.35942 | 0.833854 | −0.48892 | 0.179408 | −0.50086 | 0.907304 | −0.24565 | 0.022882 |
| *WNT3A* | 0 | −0.67551 | −0.72677 | −0.7617 | −0.83505 | −0.71153 | −0.5822 | −0.63395 | −0.57971 | −0.63639 |
| *WNT5A* | 0 | 0.17641 | 0.221571 | 0.930185 | −0.23597 | 1.33236 | −0.18782 | 0.774853 | 0.13969 | 1.103575 |
| *WNT5B* | 0 | 0.043951 | 0.110186 | 0.737527 | −0.47978 | 1.392565 | −0.4076 | 0.636378 | −0.25166 | 1.148729 |
| *WNT6* | 0 | −0.87128 | −0.49785 | −0.19866 | −0.68152 | 0.363048 | −0.97964 | −0.81446 | −0.50752 | 0.504525 |
| *WNT7A* | 0 | −1.81816 | −0.78995 | −0.8671 | −0.60924 | 0.326909 | −0.52506 | −0.60995 | −0.51956 | 0.218536 |
| *WNT10A* | 0 | −0.37151 | −0.91328 | 0.282962 | −1.07572 | 0.938009 | −0.81107 | −0.26368 | −0.78436 | 0.986176 |
| *WNT10B* | 0 | −0.16679 | −0.49485 | −0.74065 | −1.0942 | −0.27213 | −0.537 | −0.72125 | −0.66716 | 0.031893 |
| *WNT11* | 0 | −0.26313 | −0.50086 | −0.18363 | −0.47379 | −0.12761 | −0.41061 | −0.29081 | −0.39319 | −0.1698 |
| *SFRP1* | 0 | −0.72979 | −0.66055 | −0.5388 | −0.7176 | −0.65443 | −0.72979 | −1.49757 | −0.51656 | −0.08852 |
| *GSK3B* | 0 | −0.50086 | −0.12465 | 0.018076 | −0.05839 | 0.028896 | −0.06742 | 0.058388 | −0.08911 | 0.080085 |
| *BMP4* | 0 | −0.6515 | 0.161338 | 0.60206 | −0.04034 | 0.025879 | 0.016866 | 0.726686 | 0.269116 | 0.197474 |
| *BMP6* | 0 | 0.329926 | −0.34132 | 0.463579 | −0.513 | 0.65805 | −0.31731 | 0.636378 | −0.51956 | 0.369067 |
| *RUNX2* | 0 | 0.287779 | −0.66959 | 0.376285 | −0.72056 | 0.28176 | −0.44977 | 0.443717 | −0.88673 | 0.17339 |
| *GREM1* | 0 | 0.661065 | 0.098124 | 1.50214 | −0.1999 | 1.515988 | 0.040958 | 1.554519 | −0.18842 | 1.055413 |
| *FRMD6* | 0 | 0.411199 | −0.11261 | 1.180037 | −0.07946 | 1.308276 | −0.09453 | 1.37089 | −0.08307 | 1.151741 |
| *KCTD11* | 0 | −0.242 | −0.16374 | 0.240824 | −0.1698 | 0.332943 | −0.06742 | 0.305244 | −0.28777 | 0.251663 |
| *SUFU* | 0 | −0.06143 | −0.19389 | 0.29199 | −0.14868 | 0.037944 | −0.13966 | 0.458774 | −0.12223 | 0.206502 |
| *FGF9* | 0 | −1.37675 | −1.34104 | −0.14448 | −1.70997 | −0.18184 | −1.39794 | −0.31489 | −2.06048 | −0.30225 |
| *OTX2* | 0 | −1.88606 | 0.191451 | −2.24413 | 0.326909 | −2.33724 | 0.046963 | −0.85387 | 0.311266 | −2 |
| *ZIC2* | 0 | −1.29243 | 0.290791 | −1.61439 | 0.263707 | −1.04287 | 0.329926 | −1.35655 | −0.23965 | −1.70115 |
| *WIF1* | 0 | 1.37366 | 0.507532 | −0.77057 | 0.706214 | −1.383 | 0.721266 | −1.28988 | 0.296204 | −1.1512 |
| *FGFR3* | 0 | −1.33161 | −0.242 | −1.33348 | −0.242 | −1.28067 | −0.23299 | −1.6038 | 0.208925 | −1.32239 |
| FOXE1 | 0 | −0.62434 | −0.72377 | −0.88807 | −0.86519 | −0.98843 | −0.71153 | −0.89585 | −0.73025 | −0.74473 |
